# New record of the fish leech *Piscicola pojmanskae* (Annelida: Hirudinida: Piscicolidae) - DNA barcoding and phylogeny

**DOI:** 10.2478/s11756-018-0081-y

**Published:** 2018-07-02

**Authors:** Joanna M. Cichocka, Aleksander Bielecki, Marek Kulikowski, Izabela Jabłońska-Barna, Katarzyna Najda

**Affiliations:** 1 Department of Zoology, Faculty of Biology and Biotechnology, University of Warmia and Mazury in Olsztyn, Oczapowskiego 5, 10-719 Olsztyn, Poland; 2 XI High School in Olsztyn, Kołobrzeska 9, 10-444 Olsztyn, Poland; 3 Department of Invertebrate Zoology and Parasitology, Faculty of Biology, University of Gdańsk, Wita Stwosza 59, 80-308 Gdańsk, Poland; 4 Department of Tourism, Recreation and Ecology, Faculty of Environmental Sciences, University of Warmia and Mazury in Olsztyn, Oczapowskiego 5, 10-719 Olsztyn, Poland

**Keywords:** Fish, Parasites, *Piscicola pojmanskae*, Hirudinea, Grayling, Sea trout

## Abstract

The aim of this study was to confirm the taxonomic status of *Piscicola pojmanskae* Bielecki, 1994 found on Salmonidae fish. The fish leech was identified based on a diligent morphological analysis as well as COI gene sequence (DNA barcoding). The phylogenetic relationship with other piscicolid leeches was analyzed as well. *Piscicola pojmanskae* was found on various fins of both graylings and the resident form of trouts. The prevalence of infection was 1.63%. In this paper, probable causes of the lack of relation between the number of leeches on the fins and the fish body length as well as the host-searching strategy used by *P. pojmanskae* are discussed.

## Introduction

Notwithstanding numerous publications on the leeches parasitizing freshwater fish, data concerning the occurrence of leeches on Salmonidae fish are relatively limited (Bielecki 1997; Kulikowski and Rokicki 1998; Jueg et al. 2004; Bielecki et al. 2011b, c, 2012, 2014; Cios et al. 2012; Kaygorodova et al. 2012). Nevertheless many species of the genus Piscicolidae parasitizing on Salmonidae have been described (Bielecki 1997; Bielecki et al. 2011b, c, 2012, 2014; Cios et al. 2012). The main hosts of piscicolids are: salmon *Salmo salar* L.,1758, sea trout *S. trutta* morpha *trutta* L., 1758, sea trout *S. trutta* morpha *fario* L.,1758, rainbow trout *Oncorhynchus mykiss* (Walbaum, 1792), thymallid fish (Thymallinae), European grayling *Thymallus thymallus* (L., 1758) (Bielecki et al. 2011b). The leeches can be found on fins and the body of their host (Bielecki 1997; Bielecki et al. 2011b, c, 2012, 2014). So far, among the piscicolids collected from Salmonidae were *Piscicola annae* Bielecki, 1997, *Piscicola siddalli* (Bielecki et al., 2012), *Piscicola witkowskii* Bielecki, 1997, *P*. *pomorskii* Bielecki, 1997, *P. elishebae* Bielecki, 1997*, P. niewiadomskae* Bielecki, 1997 and *P. kusznierzi* Bielecki, 1997, *Cystobranchus respirans* (Troschel, 1850) (Bielecki 1997; Bielecki et al. 2011a, b, 2012; Soes 2014). Interestingly, until now the most common fish parasite *Piscicola geometra* (L., 1753) has not been recorded on salmonid fish. In the literature, there is a limited information on the influence of infection with leeches on the length, weight and condition of fish even though. According to observations of anglers, fish infected with a large number of leeches have a poorer condition. Fish leeches feeding on blood, for example *P. geometra* or *P. pojmanskae*, stay on their hosts for a comparatively short time – up to 24 h. Since leeches drop off quickly after attaching to a host, parasitologists have shown little interest in studying them (Utevsky and Trontelj 2004). However, *C. respirans* stays on its host throughout its life cycle (about 3–4 months) and leaves the host only in order to lay cocoons. Some parasitic leeches can occur in huge amounts. A good example is *Johanssonia kolaensis* (Selensky, 1914), which parasitizes *Anarhichas* spp. fish and destroys their fins in extreme cases (Epshtein 1966). Moreover, piscicolids are vectors of fish haematozoans (Khan and Paul 1995). Only in a few publications, authors analyze the relation between fish and leeches parasitizing them (Bielecki 1977, 1978, 1988a, b). In recent years, such relations were described in reference to the invasion of *C. respirans* locating itself mainly on fins of European grayling *T. thymallus* (Bielecki 1997; Bielecki et al. 2011b) and *Caspiobdella fadejewi* Epsthein, 1961 parasitizing atlantic sturgeon *Acipenser oxyrinchus* Mitchill, 1815 (Bielecki et al. 2011c). In waters belonging to the catchment of the Baltic Sea there have been recorded following fish leeches parasitizing Salmonidae fish: *Piscicola pomorskii*, *P. annae*, *P*. *elishebae*, *P*. *niewiadomskae*, *P*. *witkowskii*, *P*. *kusznierzi* (Bielecki 1997). We put forward the hypothesis that most probably among leeches recorded on fish collected from the Reda River the species mentioned above should be expected. As the morphological features of piscicolid leeches can be misleading during the identification we incorporated DNA barcoding to establish taxonomic status of collected leeches.

The piscicolid leeches that have ocelli on the posterior sucker are characterized by positive phototaxis (Bielecki 1988a, b, 1997, 1999; Bielecki et al. 2011b). Since the only leech species of piscicolids examined by Herter (1936) was *P. geometra*, which appeared to be negatively phototactic, in most of monographs (Pawłowski 1936; Lukin 1976; Sawyer 1986) is clearly stated that the negative phototaxis is the only possible mechanism of directing movement in leeches. In order to find the mechanism of host-searching in leeches the main focus has been put on chemotaxis. It resulted in formulating the theory of chemotaxis which has been occurred in literature up to date (Friesen 1981; Khan and Emerson 1981). The first attempt to connect positive reaction to the light with a host of leeches was emphasized by Bielecki (1999). It was clearly shown that *C. respirans* and *P. geometra* find their fish hosts by reaction for their shadow, and in this case positive phototaxis is a pivotal condition (Bielecki 1988a). In most fish leeches main photoreceptors are eyes and ocelli. Eyes are localized on the anterior sucker as one or two pairs of eyespots, in some species there can be third pair on a trachelosome (Sawyer 1986; Bielecki 1997). Ocelli occur in the number of eight to 16 (depending on species) on a posterior sucker, as well as in some species they are arranged in one or two lines on the sides of urosome (Sawyer 1986; Bielecki 1997).

These leeches were recorded frequently on the pelvic and pectoral fins of fish as they afford the easiest access for these parasites as they are the closest to the river bottom. Moreover, the pelvic and pectoral fins, which are outstretched laterally, offer a relatively large space. In addition, the pelvic and pectoral fins are located in the middle of the body, which is also significant. In our study we intended to check if the fish leeches localize on their host in the manner confirming assumptions of the host-searching mechanism based on positive phototaxis.

## Material and methods

In total, 757 graylings *T. thymallus*, 349 sea trouts *S. trutta* resident form and 9 sea trouts *S. trutta* anadromous form were caught from September 2012 to March 2014 with a fly fishing rod in the Reda River near Wejherowo (58° 37′ 00.00“ N, 18° 13’ 20.00” E) (Table 1). The fishes were released alive immediately after collecting leeches.

**Table 1:** Number and length [mm] of sea trout *S. trutta* resident (R) and anadromous (A) forms, and grayling *T. thymallus*. (+) the presence of *Piscicola pojmanskae*

Date	Sea trout					Grayling				
	R				A					
	<330 mm		>330 mm	Total	>450 mm	<350 mm		>350 mm		Total
Sep 2012	65		7	72	3	28		6		34
Oct 2012	12		2	14	0	46		8		54
Nov 2012	7		1	8	0	57		11		68
Dec 2012	9		9	18	0	77	+	9		86
Jan 2013	27		4	31	1	26		8		34
Feb 2013	16	+	2	18	2	62		12		74
Mar 2013	35	+	2	37	3	62	+	14		76
Apr 2013	36	+	3	39	0	51		11		62
May 2013	25		2	27	0	41		6		47
Jan 2014	1	+	16	16	0	24		62		86
Feb 2014	33	+	6	40	0	52		38	+	90
Mar 2014	26		3	29	0	37		9		46
Total	292		57	349	9	563		194		757

A number of leeches were recorded on all fins and on the trunk. On grayling there were found five leech specimens whereas on sea trout – 15 leeches (Table 2). Information on newly collected material as well as additional material (other representatives of the genus *Piscicola*, as well as *C. respirans*, *C. fadejewi* and *E. octoculata* and *G. concolor* as an outgroup) included in this study, with the locality and GenBank accession numbers is presented in Table 3.

**Table 2:** The parasitological parameters of grayling (*T. thymallus*) and sea trout (*S. trutta*) resident form infected by *P. pojmanskae*

Species	Number of fish examined	Number of fish infected	Number of parasites	Prevalence [%]	Mean intensity	Range of intensity	Abundance
*T. thymallus*	757	5	5	0.66	1.00	1–1	0.01
*S. trutta*	349	13	15	3.72	1.15	1–2	0.04
Total	1106	18	20	1.63	1.11	1–2	0.02

**Table 3:** Collection localities and GenBank accession numbers for species used for the phylogenetic analysis

Taxon (locality)	GenBank accession number
*Erpobdella octoculata*	AF003274
*Glossphonia concolor*	AY962458
*Branchellion torpedinis* (USA)	AF003265
*Johanssonia arctica* (Canada)	DQ414320
*Caspobdella fadejewi*	AY336020
*Cystobranchus respirans* (Slovenia)	AY336021
*Cystobranchus respirans* (Poland)	–
*Piscicola geometra* (France)	AF003280
*Piscicola geometra* (Germany)	AY336014
*Piscicola geometra* (Poland, Ukiel)	–
*Piscicola geometra* (Ukraine)	AY336015
*Piscicola* cf. *annae* (Germany)	AY336014
*Piscicola milneri* (Canada)	DQ414337
*Piscicola pojmanskae* (Poland, Reda - *Salmo*)	MH395321
*Piscicola pojmanskae* (Poland, Reda - *Thymallus*)	MH395320
*Piscicola* sp. (Germany)	AY330617

The leeches were collected from fish and preserved in 70% or 96% ethanol (for morphological and molecular analyses respectively), and then the fish were measured and released into the wild. The fish were divided according to their length into two classes, below or above the minimum landing size: the total length of 330 mm for the trout resident form, 450 mm for the anadromous form and 350 mm for the grayling. The taxonomic status of piscicolid representative could not be determined for certain on the basis of morphological features. Hence, the taxonomic placement was confirmed on the basis of molecular analyses of COI gene sequence.

For molecular studies, leeches were narcotized using an ethanol gradient, adding a few drops of 96% ethanol to a plastic container with water covering the leeches until they were relaxed. Specimens then were transferred to 96% ethanol for storage.

Leech tissue samples were obtained from the posterior sucker of the leech in order to minimize the possibility of contamination from host DNA that could be present in the gastro-intestinal tract. DNeasy Tissue Kit (Qiagen, Valencia, CA) was used for tissue lysis, total DNA extraction and purification. The mitochondrial cytochrome c oxidase subunit I (COI) was obtained using the primers LCO1490 and HCO2198 (Folmer et al. 1994) and used to investigate the internal relationships of the genus. Amplification reactions of each gene fragment were conducted using 0.5 μl of each 10 μM primer, 1 μl DNA template, and 23 μl Rnase-free H_2_O (total volume of 25 μl). PCR reactions were performed with an Eppendorf Mastercycler (Eppendorf, Hamburg, Germany). The following amplification protocols were used: 94 °C (5 min), followed by 30 cycles of 94 °C (45 s), 48 °C (45 s), 72 °C (1 min) and final extension of 72 °C (7 min). Sequencing reactions were performed by Genomed S.A. (Warsaw, Poland). The obtained sequence were deposited with INSDC under the accession numbers: MH395320 for *P. pojmanskae* collected from graylings and MH395321 for *P. pojmanskae* collected from trout. To verify quality of sequencing, the BLAST searches were performed.

Complimentary strands were reconciled using Sequencher 5.4 (Gene Codes Corporation). Alignment of COI sequences was performed using Clustal W with parameters of 10: 5 for the pairwise alignment and 10:5 for the multiple alignment in MEGA version 6 (Tamura et al. 2013).

Bayesian Inference was conducted in BEAST v.1.8.3 (Drummond et al. 2012). HKY model of evolution was applied based on the option Find Best DNA Model in the program MEGA 6. For the MCMC analysis, default prior distributions of parameters were used for 5 million generations, and trees were sampled every 1000 generation. The program Tracer v.1.6 (Rambaut et al. 2014) was used for visual inspection and summarizing the posterior estimates of the various parameters sampled by the Markov Chain. The maximum clade credibility tree was obtained using TreeAnnotator v.1.8.3 (Rambaut and Drummond 2012) and posterior probabilities were shown as mean heights. The obtained tree was explored in FigTree v.1.4.2 (Rambaut 2014).

The species status was confirmed by K/theta ratio (Birky et al. 2010; Birky 2013). In the COI phylogeny of *Piscicola*, well-supported clades (with posterior probabilities above 0.85) were identified, which could represent different species following the evolutionary genetic species concept (Birky and Barraclough 2009). As all pairwise sequences differences were small we used the uncorrected sequence difference D and calculated D/θ as a close approximation to K/θ (Birky 2013).

Only *P. pojmanskae* was taken into account in statistical analyses. The results concerning both species of fish, i.e. the grayling and salmon, were combined due to a low number of specimens collected. All statistical procedures were performed with STATISTICA 12.0. Non-parametric tests (χ^2^ 2 × 2 tables of frequencies and Spearman’s rank correlation coefficient) were used because the distribution of parasites deviated from the normal. Spearman’s rank correlation coefficients were calculated for relations between the body length of parasitized fish species and the number of leeches that were located on fins.

## Results

**Fig. 1:**
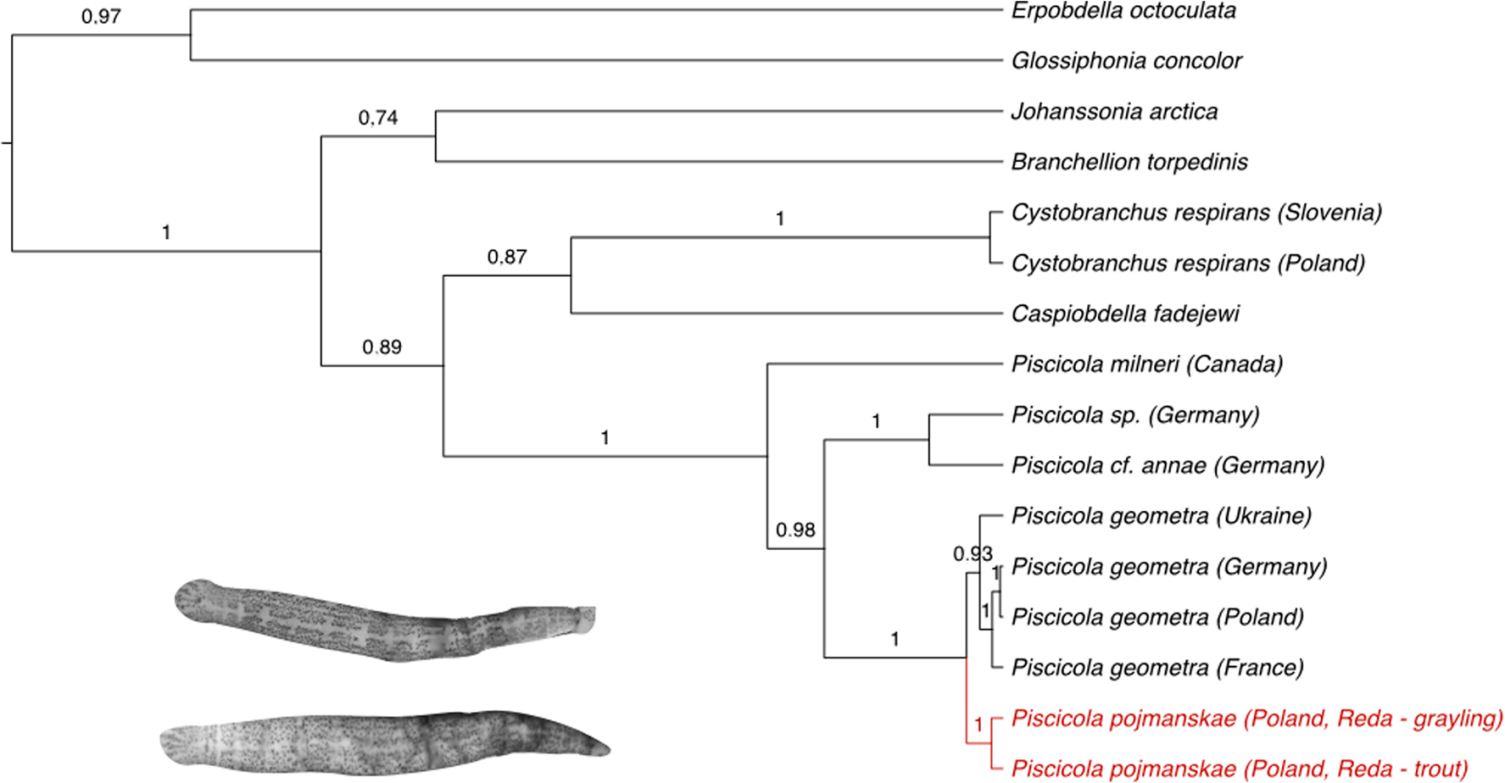
Phylogenetic position of *Piscicola pojmanskae* within piscicolid leeches. Result of Bayesian inference based on COI gene sequences; number above branches indicate the posterior probabilities

**Fig. 2:**
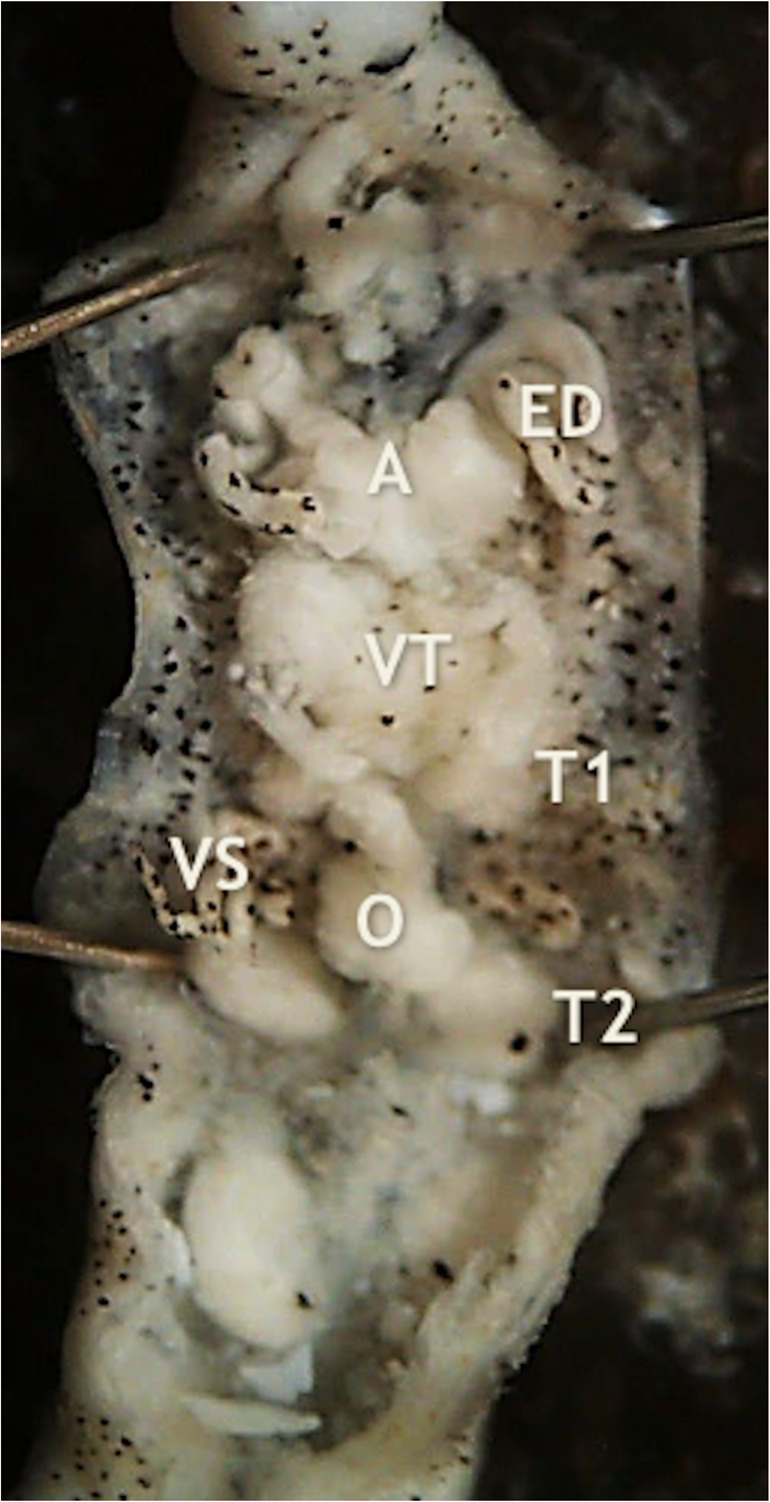
The structure of the reproductive system of *P. pojmanskae*: A – atrium, ED – ejaculatory ducts, O – ovisacs, T_1_, T_2_ – first and second pair of testisacs, VS – seminal vesicles, VT – vector tissue

In the collected material, three species of leeches were identified: *P. pojmanskae* (20 specimens), *Erpobdella octoculata* (L., 1758) (one specimen) and *Glossiphonia concolor* (Apáthy, 1888) (one specimen) (Table 2)*.* In this study, the COI gene sequence for *P. pojmanskae* is used for the first time. Bayesian inference performed using this sequence positioned *P. pojmanskae* in the separate clade within the monophyletic group comprising representatives of other considered *Pisciola* species, including *P. geometra,* from different localities (mean distance = 0.118, posterior probability = 1; Fig. 1). Details of external and internal morphology, especially the structure of the reproductive system (Fig. 2), seem to be relevant characters for the determination of *P. pojmanskae* according to its description by Bielecki (1994, 1997). Moreover, D/θ ratio was above 4 (D/θ = 4, Table 4), which according to the „4 × rule” confirmed the species status of considered fish leech.

**Table 4:** D/theta ratio calculations for *P. pojmanskae* - *P. geometra* clade COI sequences

d	π	θ	D	D/theta	n^1^, n^2^
0.003	0.005	0.005	0.02	4	2, 4

*d* mean pairwise difference, *π* nucleotide diversity, *θ* population genetic parameter theta, indicating genetic variability within populations. *D* genetic distance between sister clades, *n*^*1*^, *n*^*2*^ number of sequences for each sister clade, θ and D were calculated as *p* distances

*Piscicola pojmanskae* was the species recorded on various fins of both grayling and resident form of trout. There were no leeches on 9 specimens of the studied anadromous form of trout. The leech recorded on the fins of 0.66% of graylings ranged from 26 to 39.6 cm, and on the fins of 3.72% trout resident form ranged from 23 to 32 cm (Table 2).

**Fig. 3:**
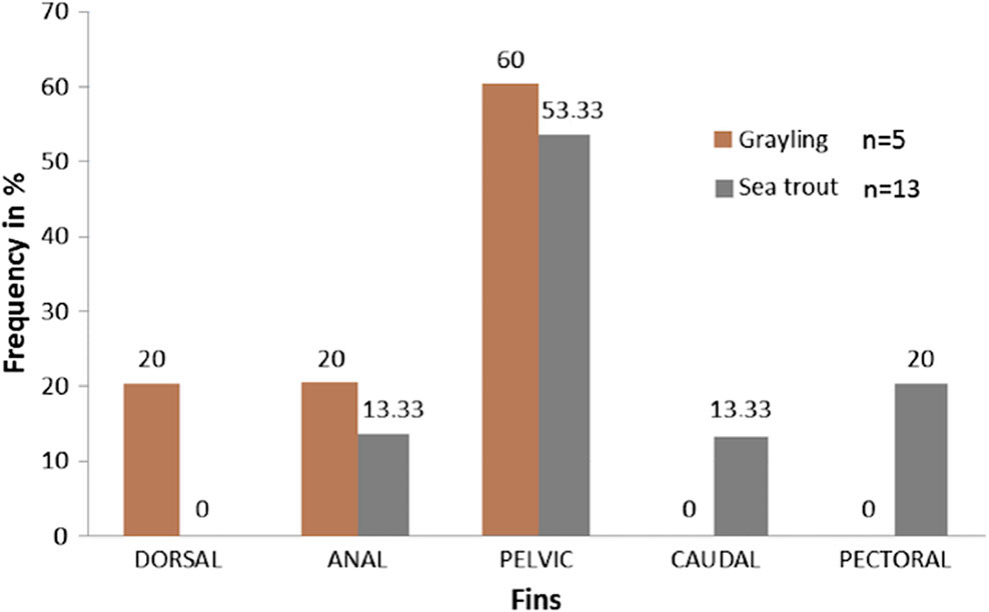
Frequency of occurrence of leech *Piscicola pojmanskae* on grayling (*T. thymallus*) and sea trout (*S. trutta*) resident form on fins, n – number of fish with leeches

For *T. thymallus,* the leech specimens were noted on dorsal, anal and pelvic fins, while for *S. trutta* resident form, the leeches occurred on all fins, except the adipose and dorsal fin. No leeches were found on the trunk of fish. In the 18 specimens of fish on which the leeches were recorded, pelvic fins were infected the most (55.56%) (Fig. 3).

Most of the fish (χ^2^, df = 1, *p* < 0.05) had only one fin infected, and only 2 trouts resident form had 2 leeches on the fin (Table 2). Statistically, one or two leeches were found in 84.62% and 15.38% of resident form of trouts, respectively (χ^2^, df = 1, *p* < 0.05).

There was no significant relation between the number of leeches on the fins and the body length of the analyzed fish (correlation coefficient *r* = −0.1313).

One specimen of *G. concolor* and one specimen of *E. octoculata* were recorded together on a grayling on a fresh wound inflicted probably by a pike. Moreover, *E. octoculata* specimen was found on a freshly healed wound located at the base of dorsal fin of another grayling (the length of fish: 305 mm and 295 mm respectively). The material was collected in January 2013.

## Discussion

Of the three species of leeches found on the salmonids from the Reda River, only *P. pojmanskae* is a parasitic species. *Erpobdella octoculata* and *G. concolor* are considered to be predatory species. Until now *P. pojmanskae* has been recorded on carp *Cyprinus carpio* (L., 1758), grass carp *Ctenopharyngodon idella* (Valenciennes, 1844), silver carp *Hypophthalmichthys molitrix* (Valenciennes, 1844) and big head carp *Aristichthys nobilis* (Richardson, 1845), mainly in fish ponds (Bielecki 1994, 1997). It was recorded most often on the body surface, fins and gills (Bielecki 1994, 1995; Bielecki et al. 2011d). Moreover, the specimens of *P. pojmanskae* were identified in the macrozoobenthos community in brackish water area (Jabłońska-Barna and Bielecki 2003; Kendzierska et al. 2014). This species occurs in lenitic reservoirs (lakes, ponds) however, it was recently found in lotic waters. All the more it is surprising and worth to be emphasized that a few specimens of *P. pojmanskae* were found on salmonid fish in the Reda River. We suppose that the leech is more numerous on cyprinid fish in the river as they were originally described from these hosts (Bielecki 1997). In this river rheophilic asp *Aspius aspius* (L., 1758), gudgeon *Gobio gobio* Cuvier, 1816, ide *Leuciscus idus* (L., 1758), and other cyprinid fish, as white bream *Blicca bjoerkna* (L., 1758), carp bream *Abramis brama* (L., 1758), white-eyed bream *Ballerus sapa* (Pallas, 1811), piked dogfish *Squalus acanthias* L., 1758, Amur bitterling *Rhodeus sericeus* (Pallas, 1776) were recorded, as well as pike *Esox lucius* L., 1758 and perch *Perca fluviatilis* L., 1758, and they could be a potential hosts of *P. pojmanskae*.

The hypothesis put in our study was tested based on 46 characters of internal and external morphology. Five unique features distinguish *P. pojmanskae* from *P. pomorskii*, *P. annae*, *P*. *elishebae*, *P*. *niewiadomskae*, *P*. *witkowskii* and *P*. *kusznierzi.* In *P. pojmanskae* papillae are localized on every third annulus of most of body somites whereas in *P. pomorskii*, *P. annae*, *P*. *elishebae*, *P*. *niewiadomskae*, *P*. *witkowskii* they occur on every fourth annulus of somite, and in *P*. *kusznierzi* papillae occur on all annuli of somites (Bielecki 1997). Distance between gonopores in *P. pojmanskae* consists of six annuli, whereas in *P. pomorskii, P*. *niewiadomskae, P*. *kusznierzi* it takes four annuli, in *P*. *elishebae* — three annuli, and in *P. annae* and *P*. *witkowskii* — two annuli (Bielecki 1997). The crop caeca and posterior crop caeca in *P. pojmanskae* is divided on five diverticuli, whereas *P. pomorskii*, *P*. *niewiadomskae, P*. *witkowskii*, *P*. *kusznierzi* there are four diverticuli, in *P. annae* — three diverticuli, and in *P*. *elishebae* — two diverticuli (Bielecki 1997). In *P. pojmanskae* seminal vesicles are classically U-shaped (Fig. 2), whereas in *P. pomorskii* and *P*. *witkowskii* they are multiply looped (arranged in 10 and more loops), in *P*. *elishebae* they are looped several times (up to four), in *P. annae* and *P*. *niewiadomskae* seminal vesicles appear as rods parallel to the long body axis, and finally in *P*. *kusznierzi* they are formed as rods transverse to the long body axis (Bielecki 1997). The conducting strands of vector tissue in *P. pojmanskae* are long (Fig. 2), and in other considered leech species they are short (Bielecki 1997). Those differences confirmed species status at morphological level and thus falsified our hypothesis.

In order to distinguish *P. pojmanskae* from very common fish leech *P. geometra* we perform detailed investigations of morphological features of the leeches, which resulted in recording 10 different characters listed in Table 5. Owing to a considerable variety of colors, why due to which it can be often confused with *P. geometra,* specimens of *P. pojmanskae* collected in this study needed to be analyzed in with regard to their taxonomic status using COI gene sequences. Bayesian inference positioned *P. pojmanskae* in the separate clade within the monophyletic group containing comprising representatives of other considered *Piscicola* species, including *P. geometra,* from different localities. The relationship was further highly supported by posterior probability of 1 (Fig. 1). Furthermore, D/θ = 4 confirmed that *P. pojmanskae* and *P. geometra* are different species (Table 4).

**Table 5:** Morphological differences between *P. pojmanskae* and *P. geometra*

Character	*Pisciola pojmanskae*	*Pisciola geometra*
Anterior sucker	elliptical, deep	circular, not very deep
Posterior sucker	elliptical or heart-shaped, very strongly muscled	circular, poorly muscled
Ocelli	10 ocelli	up to 12, rarely 14
Segmentation	14 unequal annuli i two groups of different length (2, 6, 11 longer)	14 unequal annuli in four groups of different length
Papillae	on every third annulus of most of the body somites	absent
Proboscis base	between ganglia I and III	at level of III ganglion
Distance between gonopores	6 annuli	5 annuli
Ovisacs	strongly twisted and coiled, polylobate, not cylindrical	elongate, cylindrical
Vector tissue	elliptical, narrow plate, transverse to the body long axis	elliptical, large plate parallel to the body long axis
Conducting strands of vector tissue	long, connect each ovisac with the mid part of a vector tissue	short, narrow, connect each ovisac with an anterior part of a vector tissue

Bielecki (1997) distinguished many species of fish leeches of which *P. annae* has been already confirmed at molecular level by Utevsky and Trontelj (2004). *P. pojmanskae* Bielecki, 1994 is a next species confirmed with DNA barcoding. It seems to stay in opposite to the hypothesis by Koperski (2017), who claims that most of the piscicolid leeches from Europe described by Bielecki (e.g. Bielecki 1997; Bielecki et al. 2011a) are forms of *P. geometra* rather than distinct species.

The occurrence of the two other species of leeches on the fish from the Reda River can be considered as unusual, as they are rarely recorded on the body of fish. *Erpobdella octoculata* is an example of predatory leeches feeding on small invertebrates (Pawłowski 1936, 1968; Bielecki 1977; Agapow et al. 2006; Koperski et al. 2008, 2011; Koperski 2009; Bielecki et al. 2011a). In the case of the studied fish, it was located near wounds, where it was probably enticed by the blood and other body fluids. Maybe it consumed tissues with blood from the wounds. Similar observations were described in the literature (Bielecki et al. 2011a; Utevsky et al. 2013). *Glossiphonia concolor* is also a predatory leech feeding on small invertebrates and sucking body fluids of molluscs (Sawyer 1986; Pawłowski 1936, 1968; Lukin 1976; Bielecki et al. 1997; Koperski 2009; Bielecki et al. 2011c). It can be assumed that it was also enticed by the blood from wounds on the body (Bielecki et al. 1997). It is worth noting that attacked, wounded fish are weaker than the others, stay at the bottom more often, and as a result, they are good specimens for studies and analyses of parasite-host and predator-prey relations.

*Piscicola pojmanskae*, similarly to *P. geometra*, shows a positive phototaxis; consequently, it assumes its resting position close to the bottom among submerged plants or on stones close to the shore (Bielecki et al. 2011a, b). As many fish leeches its occurrence should be expected on the pelvic and pectoral fins of fish. In this study, there was no significant relation between the number of leeches on the fins and the body length of the examined fish. It may result from the fact that the environment of the study – a fast flowing („mountainous”) river – is not favourable for *P. pojmanskae*. This species prefers more stagnant waters such as ponds, lakes and costal waters. Slow flowing rivers or oxbow lakes, due to an increase in the speed of flow, are a less favourable environment (Jabłońska-Barna et al. 2017). Furthermore, larger fish tend to occupy deeper areas with fast flowing water, increasing the possibility of leech detachment. In all probability, that is why the number of leeches recorded on the bigger fish in this study was lower or even the same.

Furthermore, the mechanism of host-searching could be explained by chemotaxis, however, it is likely to dominate in stagnant water (e.g., lakes, ponds), where sending and receiving chemical signals seem to be more effective. In lotic (fast flowing) waters, such as rivers and especially brooks, chemical signals are rapidly dispersed and cannot be perceived by chemoreceptors in leeches giving appropriate information about host localization. In this case, in fast flowing waters or its areas which are overexposed to the bottom, the mechanism of host-searching can be based on positive phototaxis and the chemotaxis seems to be a secondary phenomenon. When reaching a fish according to phototaxis mechanism, the leeches recognize whether they have found an appropriate host perceiving chemical signals from fish body (Bielecki 1997, 1999; Bielecki et al. 2011b, c).

It can be concluded that *P. pojmanskae* is not a species characteristic of grayling and trout, which are fast flowing water fish. Details of internal morphology, especially the structure of the reproductive system (Fig. 2), seems to be relevant characters for the determination of *P. pojmanskae.*

## Conclusions

1. The first hypothesis that among leeches recorded on fish from the Reda River the specimens following species should be expected: *Piscicola pomorskii*, *P. annae*, *P*. *elishebae*, *P*. *niewiadomskae*, *P*. *witkowskii*, *P*. *kusznierzi*, was falsified basing on results of morphological and molecular analyses. Out of the three species of leeches found on the salmonids from the Reda River, only *P. pojmanskae* is a parasitic species. *Erpobdella octoculata* and *G. concolor* are considered to be predatory species, and their occurrence on the fish can be regarded as accidental.
2. *Piscicola pojmanskae* were collected from salmonid fish for the first time and were recorded on all fins, except the adipose and dorsal fin. Therefore the distribution of leeches on trout and grayling is associated with mechanism of host-searching based on phototaxis.
3. In the presented study, no significant relation between the number of leeches on the fins and the body length of the analyzed fish has been proved. It may result from the fact that Reda, as a fast-flowing river, is not a favorable environment for *P. pojmanskae*, and is not a species characteristic of trout and grayling.
